# PI-RADS upgrading as the strongest predictor for the presence of clinically significant prostate cancer in patients with initial PI-RADS-3 lesions

**DOI:** 10.1007/s00345-024-04776-x

**Published:** 2024-02-16

**Authors:** Jeremy Kwe, Martin Baunacke, Katharina Boehm, Ivan Platzek, Christian Thomas, Angelika Borkowetz

**Affiliations:** 1 Department of Urology, University Hospital Carl Gustav Carus, Technische Universität Dresden, Fetscherstraße 74, 01307 Dresden, Germany; 2 Department of Radiology, University Hospital Carl Gustav Carus, Technische Universität Dresden, Dresden, Germany

**Keywords:** Follow-up, MRI, PI-RADS, PSA density, Prostate cancer, Prostate biopsy, Targeted biopsy

## Abstract

**Purpose:**

Unclear lesions on multiparametric magnetic resonance tomography (mpMRI) are challenging for the indication of biopsy in patients with clinical suspicion of prostate cancer (PCa). The aim of this study is the validation of the detection rate of clinically significant PCa (csPCa) in patients with PI-RADS 3 findings and to determine the appropriate follow-up strategy.

**Methods:**

In this retrospective single-center study, patients with maximum PI-RADS 3 lesions underwent targeted MRI/ultrasound-fusion biopsy (tPbx) combined with systematic 12-core biopsy (sPbx) and follow-up mpMRI with further control biopsy. We assessed the evolution of MRI findings (PI-RADS, volume of the lesion), clinical parameters and histopathology in follow-up MRI and biopsies. The primary objective is the detection rate of csPCa, defined as ISUP ≥ 2 findings.

**Results:**

A total of 126 patients (median PSA 6.65 ng/ml; median PSA-density (PSAD) 0.13 ng/ml^2^) were included. The initial biopsy identified low-risk PCa in 24 cases (19%). During follow-up biopsy, 22.2% of patients showed PI-RADS upgrading (PI-RADS > 3), and 29 patients (23%) exhibited a tumor upgrading. Patients with PI-RADS upgrading had a higher risk of csPCa compared to those without PI-RADS upgrading (42.9% vs. 9.18%, *p* < 0.05). PI-RADS upgrading was identified as an independent predictor for csPCa in follow-up biopsy (OR 16.20; 95% CI 1.17–224.60; *p* = 0.038).

**Conclusion:**

Patients with stable PI-RADS 3 findings may not require a follow-up biopsy. Instead, it is advisable to schedule an MRI, considering that PI-RADS upgrading serves as an independent predictor for csPCa.

## Introduction

Multiparametric magnetic resonance imaging (mpMRI) in the biopsy pathway has become a standard method for the detection of prostate cancer (PCa) and has shown high accuracy in detecting clinically significant PCa (csPCa) [1, 2]. National and international guidelines recommend performing mpMRI for all patients requiring prostate biopsy, regardless of whether they are biopsy-naïve or have undergone a previous biopsy [3, 4]. The EAU guideline recommends a systematic (sPbx) combined with MRI/ultrasound-fusion targeted biopsy (tPbx) in the case of tumor-suspicious lesion in mpMRI for biopsy-naïve patients and a sole tPbx for patients with prior negative biopsy [3]. Nevertheless, the management of PI-RADS 3 lesions, which are considered as equivocal, remains controversial, with conflicting findings regarding their significance [5–10]. Currently, there are no established recommendations for managing and following up on these lesions. Some studies suggest incorporating PSA-density (PSAD) values with PI-RADS category to guide biopsy decisions of whether to proceed with or postpone a biopsy [8, 11–13]. Several clinical parameters, biomarkers, and nomograms were described to assist in the biopsy decision in patients with lesions of PI-RADS ≥ 3 [14, 15]. In this study, we analyzed the follow-up mpMRI findings and histological results of patients who initially presented PI-RADS 3 lesions in initial biopsy. The analysis included monitoring the development of clinical and imaging parameters over time. The objectives of this study were to confirm the presence of csPCa through imaging and biopsy during subsequent follow-up and to identify clinical and imaging parameters that can predict csPCa during the follow-up.

## Patients and methods

This retrospective single-center study included patients with initial PI-RADS 3 lesions only, who underwent tPbx and sPbx and a further mpMRI combined with a follow-up biopsy. This study was approved by the Ethic committee of the Technische Universität Dresden (EK53022014).

Both biopsy naïve and previously biopsied patients were included in the study. Patients without tumor and patients with ISUP 1 tumors detected in initial biopsy were investigated. PI-RADS, number of lesions, histopathological results, clinical parameters and the risk calculator (RC) based on European Randomized study of Screening for Prostate Cancer (ERSPC) between initial and follow-up mpMRI and biopsy were compared [16]. The RC used for this study are the RC2 and RC3 from ERSPC. The RC2 is a PSA-based RC that examines the PSA value to predict the need for further testing. The RC3 was developed to calculate the risk of PCa detectable by biopsy. The result is expressed as a percentage in any PCa risk (anyERSPC3) and csPCa risk (signERSPC3). The anyERSPC3 assists in prostate biopsy decision making. A prostate biopsy is recommended if the result is 20% or more and no prostate biopsy if the result is less than 12.5%. The signERSPC3 calculates the probability of csPCa (http://www.prostatecancer-riskcalculator.com/). In our study, a PCa with Gleason Score ≥ 7 (3 + 4) or International Society of Urological Pathology (ISUP) ≥ 2 was defined as csPCa.

Primary objective was the detection rate of csPCa in patients with PI-RADS 3 lesions in the initial and follow-up biopsy. Further objective was to evaluate, which clinical and imaging parameters are eligible as predictors for csPCa. The development of the PI-RADS finding and clinical parameters between initial and follow-up biopsy in the context of histopathologic results were analyzed.

The mpMRI was performed at the Institute for Diagnostic and Interventional Radiology at the Technische Universität Dresden using a 3T MRI system (Siemens Medical Solutions, Erlangen, Germany) without an endorectal coil or external radiological practices. The mpMRI followed the ACR guidelines, including T1-weighted, T2-weighted, diffusion-weighted, and perfusion-weighted sequences, evaluated according to PI-RADS criteria. Transperineal tPbx and sPbx were conducted in the Department of Urology using the BioJet system (dk Technologies, Barum, Germany) and bkfusion systems (bk, Herlev, Denmark). In tPbx, at least 2 cores were taken per lesion based on size. Statistical analysis employed SPSS v26.0 (IBM Corp, Armonk, NY, USA), reporting continuous variables using mean, standard deviation, median, interquartile range (IQR), and minimum or maximum values. The McNemar test assessed csPCa detection rate between biopsy methods (*p* ≤ 0.05). Receiver operating characteristic (ROC) analysis, calculating the area under the curve (AUC), compared clinical parameters' significance in detecting csPCa. Uni- and multivariate binary logistic regression analysis identified predictors for PCa or csPCa presence based on clinical and imaging parameters. Therefore, continuous parameters were dichotomized according the median or the clinical impact. Using a Sankey diagram, the course between the initial and follow-up PI-RADS is presented depending on the biopsy result.

## Results

**Fig. 1:**
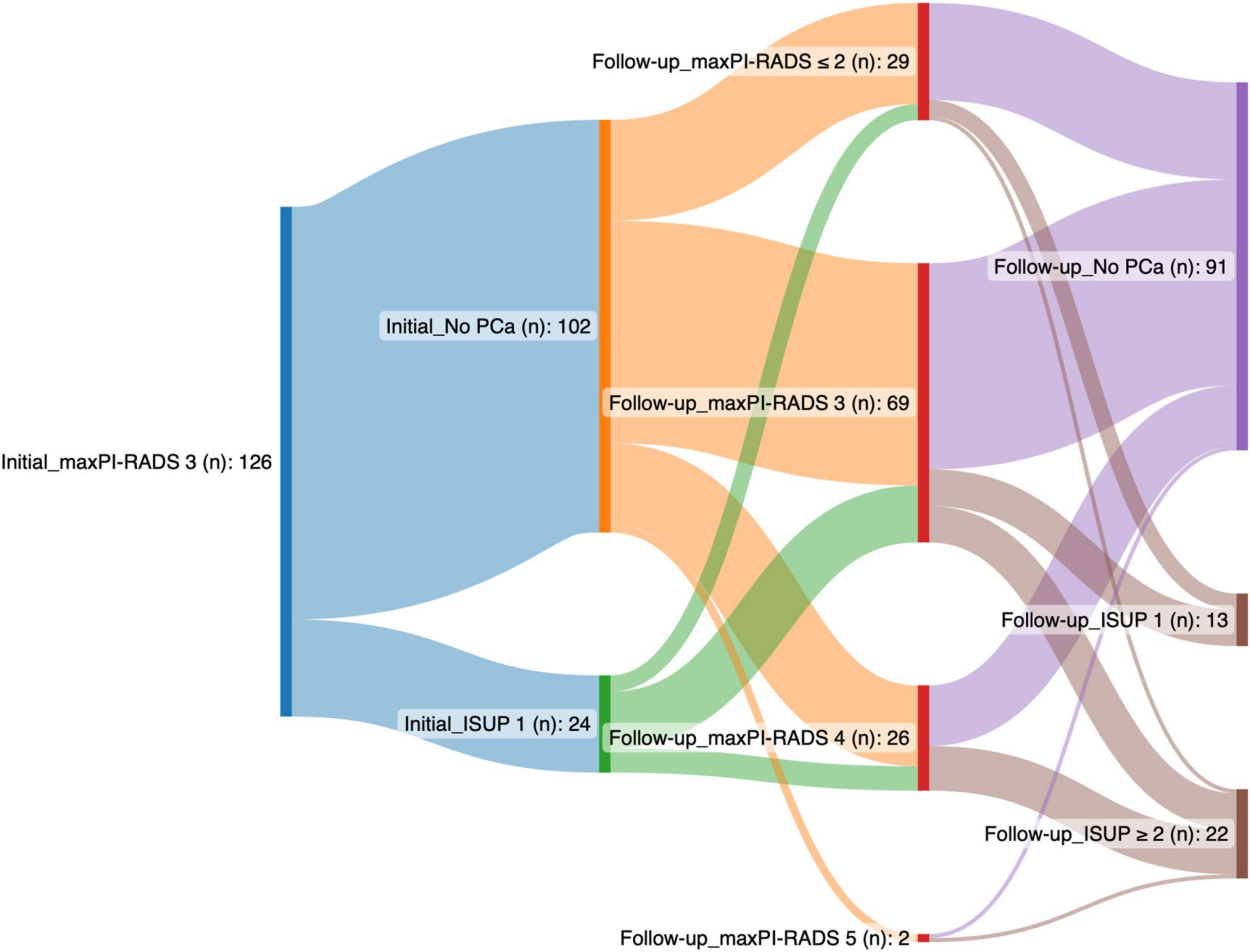
Course of change (n) in maxPI-RADS between the initial and follow-up biopsy

Between January 2016 and October 2022, 126 patients with a maximum PI-RADS 3 lesion underwent initial Pbx (30 patients with primary biopsy, 96 patients with repeat biopsy). 19% (24/126) presented PCa with ISUP 1. sPbx detected more PCa compared to tPbx (83% vs. 54%; *p* < 0.001). All patients underwent follow-up biopsy at our department (Table 1). The interval between initial and follow-up biopsy was 23 months. During this time 28 patients showed a PI-RADS-upgrading, 26 with PI-RADS 4 and 2 with PI-RADS 5 (Fig. 1). In the follow-up biopsy 35 patients had PCa, whereas 21 patients (60%) had a csPCa (ISUP 2:n = 19, ISUP ≥ 4: *n* = 2). 23% of patients (29/126) had a downgrading of PI-RADS and a stable MRI-finding with a PI-RADS 3 was found in 54.8% of patients (69/126). CsPCa was detected in 3.4% of men with PI-RADS < 3, 11.6% of men with PI-RADS 3, 42.3% of men with PI-RADS 4, and 50% of men with PI-RADS 5 findings (*p* < 0.001). In the follow-up biopsy, sPbx detected more csPCa compared to the tPbx for patients with PI-RADS 3 and 4 (PI-RADS 3: 63.6% (7/11) vs. 45% (5/11); *p* < 0.001, PI-RADS 4: 90.9% (10/11) vs. 63.6% (7/11); *p* = 0.002). For PI-RADS 5, both sPbx and tPbx detected an equal amount of csPCa with 100% (1/1; *p* = 0.16).

**Table 1:** Description of the study cohort

	Initial biopsy	Follow-up biopsy
Age (years)		
Median (IQR)	63 (59–68)	65 (61- 70)
Total PSA (ng/mL)		
Median (IQR)	6.65 (4.78–9.20)	7.39 (5.43–11.53)
PSA density (ng/mL^2^)		
Median (IQR)	0.13 (0.08–0.18)	0.14 (0.10–0.22)
Prostate volume (mL)		
Median (IQR)	67 (44.5–81.55)	68 (35–75)
Repeat biopsy (*n* (%))	96 (76.2%)	126 (100%)
Number of previous biopsies (*n*)		
Median (IQR)	1 (1–2)	2 (1–3)
Active surveillance (*n* (%))	19 (15.1%)	36 (28.6%)
Number of lesions (*n*)		
Median (IQR)	2 (1 -2)	1 (1–2)
Suspicious digital-rectal examination (*n* (%))	8 (6.35%)	9 (7.14%)
ERSPC2		
Median (IQR)	31,5 (25 -39.25)	34 (27–45)
anyERPSC3		
Median (IQR)	8 (6 -12.5)	10 (6–22)
signERSPC3		
Median (IQR)	2 (2–4)	3 (1–6)
PI-RADS ≤ 2		29 (23%)
PI-RADS 3		69 (54.8%)
PI-RADS 4		26 (20.6%)
PI-RADS 5		2 (1.6%)
Detection of PCa (*n* (%))		
ISUP 1	24 (19%)	14 (11%)
ISUP2		19 (15.1%)
ISUP ≥ 4		2 (1.6%)
ISUP upgrading (*n* (%))		
Overall		29 (23%)
ISUP 1		8 (27.6%)
ISUP 2		19 (65.5%)
ISUP ≥ 4		2 (6.9%)
Number of inflammation (*n* (%))	28 (22%)	23 (18.3%)
ISUP downgrading (no PCa) (*n* (%))		9 (7.1%)

In the ROC analysis, the PSAD showed the highest accuracy comparing to other parameters in detecting csPCa with an AUC of 0.546 (*p* = 0.511; 95% CI 0.410–0.681). ERSPC3 for the detection of any PCa discriminated csPCa with an AUC of 0.540 (*p* = 0.561; 95% CI 0.406–0.648). PSA and ERSPC2 showed a lower AUC of 0.512 (*p* = 0.861; 95% CI 0.376–0.648). ERSPC3 for the detection of csPCa came last with an AUC of 0.455 (*p* = 0.515; 95% CI 0.321–0.589). In the uni- and multivariate binary logistic regression analysis, the upgrading of PI-RADS to 4 was the only independent predictor for the detection of csPCa (Table 2).

**Table 2:** Binary logistic regression analysis of clinical and imaging parameters to predict csPCa in initial and follow-up biopsy (OR—odds ratio; CI—confidence interval), significant parameters in multi-variate analysis ist depicted in bold

	Uni-variate analysis				Multi-variate analysis			
	OR	95% CI	*p* value	OR	95% CI	*p* value	OR	95% CI
Initial biopsy								
Age < vs. ≥ median (63 years)	1.930	0.738	5.044	0.180	–	–	–	–
PSA < vs. ≥ median (6.65 ng/ml)	1.121	0.439	2.864	0.811	–	–	–	–
Prostate volume < vs. ≥ median (67 ml)	0.800	0.297	2.152	0.659	–	–	–	–
PSAD < vs. ≥ median (0.13 ng/ml^2^)	1.442	0.563	3.693	0.445	–	–	–	–
PSAD < vs. ≥ 0.15 ng/ml^2^	1.324	0.501	3.502	0.572	–	–	–	–
PSAD < vs. ≥ 0.20 ng/ml^2^	2.200	0.742	6.519	0.155	–	–	–	–
Number of lesion	1.414	0.461	4.337	0.544	–	–	–	–
Previous non-csPCa no vs. yes	1.412	0.417	4.775	0.579	–	–	–	–
Number of previous biopsies < vs. ≥ 1	1.048	0.371	2.963	0.929	–	–	–	–
ERSPC2 < vs. ≥ median (31.5%)	1.122	0.439	2.868	0.810	–	–	–	–
ERSPCany3 < vs. ≥ median (8%)	1.393	0.533	3.643	0.499	–	–	–	–
ERSPCsign3 < vs. ≥ median (2%)	0.691	0.264	1.806	0.451	–	–	–	–
Time interval < vs. ≥ median	1.851	0.711	4.853	0.207	–	–	–	–
PI-RADS upgrading								
PI-RADS 3 no vs. yes	3.672	0.438	30.791	0.231	–	–	–	–
PI-RADS 4 no vs. yes	**20.533**	**2.413**	**174.700**	**0.006**	**16.202**	**1.169**	**224.599**	**0.038**
PI-RADS 5 no vs. yes	28.000	0.921	851.593	0.056	–	–	–	–
Follow-up biopsy								
Age < vs. ≥ median (65)	2.468	0.921	6.612	0.072	–	–	–	–
PSA < vs. ≥ median (7.39 ng/ml)	1.079	0.422	2.760	0.874	–	–	–	–
Prostate volume < vs. ≥ median (68 ml)	**0.214**	**0.059**	**0.775**	**0.019**	0.331	0.068	1.610	0.171
PSAD < vs. ≥ median (0.14 ng/ml^2^)	**5.714**	**1.783**	**18.309**	**0.003**	3.595	0.249	51.854	0.347
PSAD < vs. ≥ 0.15 ng/ml^2^	**5.053**	**1.701**	**15.010**	**0.004**	1.419	0.084	24.066	0.808
PSAD < vs. ≥ 0.20 ng/ml^2^	**3.395**	**1.268**	**9.088**	**0.015**	0.810	0.169	3.874	0.792
Number of lesion	0.488	0.166	1.434	0.192	–	–	–	–
ERSPC2 < vs. ≥ median (34%)	1.079	0.422	2.760	0.874	–	–	–	–
ERSPCany3 < vs. ≥ median (10%)	**5.743**	**1.928**	**17.107**	**0.002**	1.487	0.107	20.628	0.767
ERSPCsign3 < vs. ≥ median (3%)	**5.743**	**1.928**	**17.107**	**0.002**	1.012	0.071	14.336	0.993

## Discussion

In the present study, the value of mpMRI and tPbx compared to sPbx in PCa detection was examined and compared in a total of 126 men with an initial maximum PI-RADS of 3. The majority of patients (76.2%) had already received at least one pre-biopsy. In the initial biopsy, PCa with ISUP 1 was found in 19% patients. Several studies have shown a variety of the detection rate of PCa for the PI-RADS 3 between 29.7% and 43.9% and for csPCa between 4 and 24.9% [13, 17–20]. The likelihood of PI-RADS 3 lesions associated with csPCa is considered rather low [5, 6, 20]. Many authors interpret PI-RADS 3 lesions as a gray zone for the detection of csPCa [7], in which the presence of a csPCA is not clearly described [2, 5]. There is still a considerable detection rate of csPCa at 14% [21], as we also demonstrated in our cohort (11.6%). Additionally, it’s important to acknowledge that in a re-biopsy cohort, the overall PCa-detection rate might be lower when compared to a primary biopsy cohort. Within our study population, patients with PI-RADS 3 lesions in the initial biopsy showed the presence of PCa with ISUP 1 in 19% of cases, which decreased to 14.5% in the follow-up biopsy. Moreover, 40% of patients exhibited signs of inflammation consistent with chronic prostatitis.

Thus, the importance of PI-RADS 3 lesion, seems to be contradictory in some works [17, 19, 20]. Considering the frequency of occurrence of PI-RADS 3 lesions, depending on the patient cohort, studies have shown that 18–46% of all mpMRI show PI-RADS 3 lesions [22, 23], as can be calculated from this work with 54.7% in the follow-up mpMRI. This relatively high number poses a significant challenge for clinical management [2, 23].

Current guidelines recommend combined biopsy for biopsy-naïve patients with PI-RADS 3 and solely tPbx for patients with previous negative biopsy [3, 4]. In our follow-up MRI after approximately 2 years, 22.2% of patients showed PI-RADS upgrading, while 23% had PI-RADS downgrading. We found an association between PCa or csPCA presence and PI-RADS progression. The detection rate was 42.3% for PI-RADS 4 and 50% for PI-RADS 5 lesions, respectively. Boschheidgen et al. showed similar results. Of 89 patients with initially maxPI-RADS 3, 19 had PCa, of which 4 cases were csPCa. After the time interval of 12–24 months, PI-RADS upgrading was evident in the follow-up MRI, especially in the patients with PCa at initial biopsy. In patients with a negative initial biopsy, there was even a significant PI-RADS downgrading. Thus, Boschheidgen et al. recommended that patients with PI-RADS 3 primarily receive a follow-up MRI within 12–24 months after the initial MRI instead of a prompt biopsy [20]. Limitation of the study by Boschheidgen et al. was that there was no follow-up of the biopsy, so that an upgrading or downgrading of the histological findings cannot be retraced. In our study, ISUP upgrading was observed in 23% of patients, with ISUP 1 in 27.6%, ISUP 2 in 65.5%, and ISUP ≥ 3 in 6.9% of cases. ISUP downgrading occurred in 7.1% of patients. In this case, the majority of patients with tumor upgrading had an ISUP ≤ 2. Our results align with other studies advocating for follow-up mpMRI to avoid unnecessary biopsies [8, 9, 17, 20, 24]. In case of a repeat biopsy, we recommend performing a combined biopsy. In this study, both sPbx and tPbx were evaluated. In the follow-up biopsy, the detection rate of sPbx was significantly higher for csPCa compared to tPbx for PI-RADS 3 and 4 (PI-RADS 3: 63.6% vs. 45%, p < 0.001; PI-RADS 4: 90.9% vs. 63.6%, p = 0.002). This may be due to the fact that the total number of patients with each associated PI-RADS lesion is relatively low. Nevertheless, this result stands in line with other studies [18, 25]. If diagnostics relied solely on tPbx, 17% of csPCa would be missed [25]. Thus, it can again be concluded that in case of a repeat biopsy the need for additional sPbx to tPbx continues to exist in the cohort with PI-RADS 3 lesions [4, 18, 25, 26].

Several studies have shown, that the additional use of the PSAD with a cut-off of 0.15 ng/ml^2^ and the risk calculator to the mpMRI contributes to a better risk discrimination for csPCa and can thus be used as a helpful decision index for the biopsy indication. In this study, the PSAD was the strongest predictor for the detection of csPCa, followed by ERSPC3. However, both PSAD and ERSPC were not independent predictors. In the uni- and multivariate analysis, the upgrading of PI-RADS to 4 was the only independent predictor for the detection of csPCa. The relatively low p value of PI-RADS upgrading to 5 is most likely due to the limited patient sample size, particularly with PI-RADS 5. Nevertheless, some studies could demonstrate, that the higher the PSAD, the more likely the probability of csPCa [27, 28]. In patients with negative MRI and PSAD > 0.15–0.20 ng/ml^2^ the risk of csPCA is up to 27–40% [8, 29, 30]. Alternatively, a biopsy can be omitted for patients with PI-RADS ≤ 3 and PSA density < 0.15 ng/ml^2^ [8, 30].

Our study has several limitations. First, it is a retrospective, single-center study that includes a heterogeneous group of patients, many of whom had undergone prior biopsies. Additionally, there was variability in the timing of follow-up MRI and biopsy among the patients. However, it is worth noting that our study achieved a realistic median time interval between the initial and follow-up MRI. Other studies have reported time frames of 12–24 months for a follow-up MRI, which reveal PI-RADS upgrading in cases of csPCa [9, 20]. It is important to acknowledge that different time intervals may lead to different outcomes, emphasizing the need for further research to determine the optimal interval. Another limitation to consider is the diversity in the MRI scans, which were performed by different physicians, both internally and externally. Furthermore, there was no secondary evaluation of the external MRI scans, which might have the potential to introduce variability in result interpretation. Moreover, for the minor group of patients with ISUP 1 PCa in the initial biopsy, we did not use the PRECISE Score to have homogenized MRI findings.

## Conclusion

Based on our findings, we suggest that patients with PI-RADS 3 findings may primarily undergo a follow-up MRI within 24 months of the initial MRI. The overall detection rate of csPCa in PI-RADS 3 lesions was relatively low. However, if there is a PI-RADS upgrade observed in the follow-up MRI, we strongly recommend a repeat biopsy, as there is a significant association between PI-RADS upgrading and PCa upgrading. Clinical parameters such as the PSAD or ERSPC3 risk calculator may provide valuable support for clinical decision-making.

## Data Availability

It was performed during the submission process.

## Abbreviations

CI: Confidence interval
csPCa: Clinically significant prostate cancer (ISUP grade ≥ 2)
IQR: Interquartile range
ISUP: International Society of Urological Pathology
mpMRI: Multiparametric prostate magnetic resonance imaging
OR: Odds ratio
PCa: Prostate cancer
PI-RADS: Prostate Imaging Reporting and Data System
PSA: Prostate-specific antigen
PSAD: Prostate-specific antigen density
sPbx: Systematic 12-core biopsy
tPbx: targeted biopsy by MRI/ultrasound-fusion biopsy
US: Ultrasound
